# The history of the research of iron in parkinsonian substantia nigra

**DOI:** 10.1007/s00702-012-0894-8

**Published:** 2012-09-02

**Authors:** Andrzej Friedman, Jolanta Galazka-Friedman

**Affiliations:** 1 Department of Neurology, Medical University of Warsaw, Warsaw, Poland; 2 Faculty of Physics, Warsaw University of Technology, Warsaw, Poland

**Keywords:** Iron, Parkinson's disease, Ferritin

## Abstract

The role of iron in pathogenesis of Parkinson’s disease is widely discussed in the literature. The authors present the history of studies of iron in parkinsonian tissue from the substantia nigra.

Iron in the brain has already been investigated in the nineteenth century. In 1886 a study on iron content in the human brain by Dr. Zaleski was published (Zaleski 1886). This author suggested that iron present in the brain is not hemoglobin bound but bound to another type of protein. Almost 40 years later, Spatz discovered high concentration of iron in “extrapyramidal centers of human brain” such as, among others, substantia nigra (SN) (Spatz 1922). In 1924 Lhermitte et al., using Perls and Turnbull staining, described an increase of the concentration of iron in globus pallidus of one patient who died with the clinical diagnosis of Parkinson’s disease (PD). Concerning the iron in SN these authors wrote explicitly that “[iron] in the substantia nigra was present in normal amounts” (Lhermitte et al. 1924). This paper is often erroneously cited as the first description of an increase in the concentration of iron in parkinsonian SN.

The quantitative studies of iron in human midbrain started with the paper by Hallgren and Sourander (1958). These authors assessed the concentration of total iron in 52 samples of human SN obtained from normal subjects. They used colorimetry and the concentration of iron in the whole SN was 184.6 ± 65.2 μg/g wet tissue (Hallgren and Sourander 1958). The first study aimed at the comparison of iron concentration in parkinsonian and control SN was that by Earle, who used X-ray fluorescent spectroscopy (Earle 1968). He examined 11 samples of parkinsonian SN obtained from formalin fixed brains and compared them to unknown number of control samples. It is important to note that the brain bank, from which he obtained the brains, kept them for extremely long time (some of them since 1862) in formalin. According to this author, the concentration of iron in parkinsonian samples was two times higher than that of control. It was only after 20 years that the first study quantitatively comparing the concentration of iron in parkinsonian SN and control was published (Sofic et al. 1988). These authors used spectrophotometry applied on small samples of 50–80 mg (eight parkinsonian and eight controls), which were pretreated by hydrochloric acid and pepsin before measurements. The concentration of iron in PD was found to be significantly higher than that in control (85 ± 11 vs. 48 ± 8 μg/g wet tissue). Two studies comparing the concentration of iron in PD and control SN, this time in lyophilized tissue, were published in the following year. One, using inductively coupled plasma spectroscopy in seven PD and nine control SN samples (Dexter et al. 1989) found a higher concentration in PD than in control (780 ± 60 vs. 580 ± 60 μg/dry tissue), while the other, in which atomic absorption and emission performed on 9 PD and 11 control samples (Uitti et al. 1989) did not find any significant difference in iron concentration (653 ± 55 vs. 613 ± 56 μg/dry tissue). Several studies dealing with iron in parkinsonian SN appeared in the following years. Griffiths and Crossman (1993) using atomic absorption for the assessment of the concentration of iron in six samples of parkinsonian and six control SNs found two times higher concentration in PD (281 ± 22 vs. 140 ± 13 μg/g wet tissue). In two subsequent studies the concentration of iron was calculated as ng/mg of protein. In the study by Mann et al. (1994) who assessed 18 PD and 22 control SNs using inductively coupled plasma spectroscopy, the ratio of iron concentration PD/control was 1.56. However, in another study published next year by Loeffler et al. (1995), who used colorimetry in 14 PD and 8 control SNs, the concentration of iron was found to be smaller in PD than in control, the iron concentration ratio PD/control being in this study 0.82. In 1996, the first assessment of the concentration of iron in SN with the use of Mössbauer spectroscopy performed in six PD and eight control SNs was published (Galazka-Friedman et al. 1996). No difference in the iron concentration between PD and control was reported. The same group of authors continued their research with the use of this method and in 2010 published the results obtained from 17 PD and 29 control SNs assessed with the use of the Mössbauer spectroscopy (Wypijewska et al. 2010). In this study no difference in the concentration of the whole iron present in SN between PD and control was found (177 ± 18 vs. 177 ± 14 μg/g wet tissue). The ratio of iron concentration in PD versus control obtained with the use of various methods is displayed in Tables 1 and 2. It is important to note that in some studies, a high error of the ratio was found suggesting a wide range of distribution of the results of the concentration of iron.

**Table 1:** Studies showing higher concentration of iron in PD substantia nigra compared to control

Author (year)	Method	No of PD samples	No of control samples	PD/control ratio
Earle (1968)	XFS	11	?	2 ± ?
Sofic et al. (1988)	SPH	8	8	1.77 ± 0.37
Griffiths and Crossman (1993)	AA	6	6	2.01 ± 0.24
Mann et al. (1994)	ICP	18	22	1.56 ± 0.69
Dexter et al. (1989)	ICP	7	9	1.34 ± 0.17

PD/control was calculated with the standard error of the ratio

*XFS* X-ray fluorescent spectroscopy, *SPH* spectrophotometry, *AA* atomic absorption, *ICP* inductively coupled plasma spectroscopy

**Table 2:** Studies showing similar concentration of iron in PD substantia nigra compared to control

Author (year)	Method	No of PD samples	No of control samples	PD/control ratio
Loeffler et al. (1995)	Col	14	8	0.82 ± 0.08
Uitti et al. (1989)	AA&E	9	12	1.07 ± 0.13
Wypijewska et al. (2010)	MS	17	29	1.00 ± 0.13

PD/control was calculated with the standard error of the ratio

*Col* colorimetry, *AA&E* atomic absorption and emission, *MS* Mössbauer spectroscopy

As seen from this review, there is no agreement in the literature concerning the ratio of iron concentration between parkinsonian and control SN. What is also surprisingis that the range of the values obtained for iron concentration in these studies is very wide—from about 40 μg/g wet tissue (Sofic et al. 1988) up to about 180 μg/g wet tissue (Wypijewska et al. 2010). If one adds the value obtained for the concentration of iron in normal SN with the use of total reflection X-ray fluorescence (about 400 μg/g wet tissue) (Zecca and Swartz 1993), the values presented in the literature differ by a factor of 10! Possible causes of this discrepancy in the results may include the methods used for the assessment, the size of the samples studied, methods of storage of samples before the assessment and the severity of the destruction of SN related to the severity of the disease.

Sofic et al. (1988) used spectrophotometry applied to samples pretreated with hydrochloric acid and pepsin. It is obvious that such a pretreatment destroys the protein shell of ferritin, where most of SN iron is located. The destruction of the protein must result in an efflux of iron, therefore the results of the subsequent assessment show only how much iron could escape from the protein during the preparation. Should there be a difference in the structure of the protein shell between PD and control making PD protein shell more susceptible for the destruction, more iron would be detected in parkinsonian samples. In fact a difference in the structure of ferritin between parkinsonian and control SN was found (Koziorowski et al. 2007). As shown in this study, the ferritin shell in parkinsonian SN contains significantly less L-ferritin compared to control (52.5 ± 8.2 vs. 97.9 ± 12.3 ng/mg wet tissue). L-ferritin is involved in iron storage potential of ferritin shell, therefore a decrease in L-Ferritin may cause an easier efflux of iron (Harrison and Arosio 1996). It is interesting that the smaller concentration of L-Ferritin compared to the control was also found in SN samples obtained from incidental Lewy bodies cases, suggesting that this change of the structure of ferritin precedes the appearance of clinical symptoms of the disease (Koziorowski et al. 2007).

The size of the sample studied may also play a role in discrepancies of the results. It has to be noticed that in most of the studies only a small part was used for determination of iron concentration with the weight of samples ranging from 10 to 60 mg. Only in the Mössbauer spectroscopy study the whole SN weighing more than 250 mg was used. It was shown that there is a difference in the concentration of iron between pars compacta and pars reticulata of SN (Sofic et al. 1991). One cannot exclude a non-homogenous distribution of iron even within pars compacta of SN.

The brain storage conditions before the assessment may also certainly play a role. The brains in the Earle study were kept for very long time in formalin, and it is known that this storage causes a leak of iron (Bauminger et al. 1994). The difference between PD and control presented in this study may, therefore, reflect the difference in the time of storage with the control brains being kept in formalin longer.

The discrepancy of the results of studies aimed to assess the concentration of the total iron in parkinsonian SN may also be related to the severity of the destruction of SN in the brain samples studied. The influence of the severity of the disease at the death of PD patient on the level of iron was suggested by Riederer et al. (1989) who found an increase in the concentration of the total iron in parkinsonian SN only in patients who died with severe and not with mild PD.

Controversies concerning iron in parkinsonian SN included also the proportion of divalent/trivalent iron. The redox state of iron was assessed in three experimental settings: with the use of spectrophotometry (Sofic et al. 1988), Mössbauer spectroscopy (Galazka-Friedman et al. 1996), and micro-XANES technique (Chwiej et al. 2007). In the first study large amounts of divalent iron were found, while neither in the second nor in the third one divalent iron was detected. The computerized simulation aimed to assess the proportion of divalent iron that could escape detection by Mössbauer spectroscopy and showed that the concentration of iron in SN cannot be higher than 5 % of the total iron (Galazka-Friedman et al. 1996). Taking into consideration the methodology used by Sofic et al. (1988), one may assume that the preparation of samples could lead to reduction of ferric to ferrous iron.

If there is no significant increase in the concentration of iron present in SN of patients who died with Parkinson’s disease, could one exclude the role of iron in pathogenesis of the disease? According to Fenton reaction only small amounts of divalent iron are necessary to trigger this process leading to the production of free radicals. In fact a recent finding of higher concentration of non-ferritin, labile iron in parkinsonian SN seems to confirm the role of iron (Wypijewska et al. 2010). It should be stressed, however, that the concentrations of this iron represent only less than 1 % of the total iron (135 ± 10 ng/g wet tissue in PD and 76.5 ± 5 ng/g wet tissue in control).

Discussing the possible role of iron, it is important to pay attention to iron-binding compounds. According to Mössbauer spectroscopy most of iron (~85 % of the total iron) is bound to ferritin (Galazka-Friedman et al. 1996). Brain ferritin is different from liver ferritin having much higher H/L ferritin ratio and also smaller iron core (Galazka-Friedman et al. 2005). It was found that in PD SN there is a substantial decrease in the concentration of L-chains of ferritin, compared to control (Connor et al. 1995; Galazka-Friedman et al. 2004). This decrease in the concentration of L-Ferritin is already detectable in preclinical stage of PD—the incidental Lewy bodies. As the L-chains of ferritin are involved in the storage of iron within the iron shell of the protein, such a decrease may lead to an easier efflux of iron from ferritin. The possible role of ferritin in PD was recently discussed (Friedman et al. 2011).

Another possible source of iron capable of triggering the Fenton reaction is neuromelanin (NM). This black pigment is abundant in substantia nigra and in locus coeruleus (LC). It possesses high ability to bind iron and therefore may chelate large amounts of it, which may suggest a possible neuroprotective role of neuromelanin in SN (Zecca et al. 2001). It was shown that neuromelanin associated redox-active iron in increased in parkinsonian SN (Faucheux et al. 2003). It was proposed that the high vulnerability of melanized dopaminergic cells of SN depends on the presence of NM (Zecca et al. 2004). On the other hand, however, the presence of NM cannot explain by itself the destruction of nervous cells in SN occurring in PD, as other highly melanized nervous cells of LC do not show such catastrophic destruction in PD. One may speculate that the destruction of SN is due to an increase of the concentration of iron above the storage possibility of NM. A release of iron from NM could trigger Fenton reaction (Zecca et al. 2004). It should be noticed, however, that although such a possibility cannot be excluded, the finding of a decreased concentration of L-ferritin in parkinsonian SN may better explain an increase of the labile iron in Parkinson’s diseases (Koziorowski et al. 2007).

In conclusion one may say that not the total iron present in the substantia nigra but only non-ferritin bound iron may be involved in pathogenesis of Parkinson’s disease. We suggest that the destruction starts with a decrease of iron storage capacity of ferritin due to a decrease of L-ferritin in the protein shell. This defect of ferritin allows some amounts of iron to leak from this protein. The iron released from ferritin enters the Fenton reaction resulting in production of hydroxyoxygen OH^·^ free radical (see below).

$$ {\text{FE}}^{2+}+{\text{H}}_{2} {\text{O}}_{2}\to{\text{Fe}}^{3+}+{\text{OH}}{\cdot} + {\text{OH}}^{-} $$

The hydroxyoxygen OH· free radical triggers the chain reactions of production of more free radicals and this process initiates neurodegeneration. Of course, one should remember that the oxidative stress injury is only one of many possible mechanisms leading to progressive destruction of nervous cells.
